# Open, laparoscopic, and robotic liver resection for hepatocellular carcinoma: evolving roles, comparative outcomes, and future perspectives

**DOI:** 10.3389/fonc.2026.1936469

**Published:** 2026-10-08

**Authors:** Jacopo Mascherini, Mira Khaldoun Eid, Garnet Vanterpool, Iswanto Sucandy

**Affiliations:** AdventHealth Tampa, Digestive Health Institute, Tampa, FL, United States

**Keywords:** hepatocellular carcinoma, laparoscopic hepatectomy, liver resection, minimally invasive liver surgery, patient selection, robotic hepatectomy, surgical oncology

## Abstract

Hepatocellular carcinoma remains one of the leading causes of cancer-related mortality worldwide and frequently arises in the setting of chronic liver disease, cirrhosis, and portal hypertension. Liver resection represents a cornerstone of curative-intent treatment, but its role is uniquely influenced by the need to balance oncologic radicality with preservation of functional liver parenchyma. Over the last two decades, minimally invasive liver surgery has progressively expanded from selected laparoscopic minor resections to complex laparoscopic and robotic hepatectomies, challenging the historical dominance of open surgery. This narrative review critically examines the evolving roles of open, laparoscopic, and robotic liver resection for hepatocellular carcinoma, emphasizing patient selection, technical considerations, scenario-based platform choice, and comparative outcomes. Particular attention is given to hepatocellular carcinoma-specific challenges, including impaired liver function, clinically significant portal hypertension, future liver remnant adequacy, recurrence risk, and the need for parenchymal-sparing strategies. The review also discusses distinct clinical scenarios, including small solitary tumors, posterosuperior lesions, central or deeply located tumors, multifocal disease, and recurrent HCC. Current evidence consistently suggests that minimally invasive approaches are associated with reduced blood loss, fewer postoperative complications, and shorter hospital stay compared with open surgery, while maintaining comparable oncologic outcomes in selected patients. Laparoscopy remains the most established minimally invasive platform, whereas robotics may offer technical advantages in complex resections through improved dexterity, three-dimensional visualization, and enhanced suturing capability. However, available evidence remains largely retrospective and influenced by selection bias and institutional expertise. Platform selection should therefore be individualized according to tumor anatomy, liver function, technical complexity, and center experience, with the goal of maximizing oncologic benefit while minimizing functional liver loss.

## Introduction

Hepatocellular carcinoma (HCC) is a major global health concern, ranking as the sixth most commonly diagnosed cancer, the third leading cause of cancer-related mortality, and the most common primary hepatic malignancy worldwide (1). Chronic hepatitis B virus (HBV) and hepatitis C virus (HCV) infections represent the most common underlying etiologies of HCC, particularly in East Asian and African countries (2, 3). However, increasing implementation of HBV vaccination protocols and antiviral HCV treatment has resulted in a decline in incidence and mortality rates of HCC (3, 4).

In contrast, other risk factors for HCC are increasing, including metabolic dysfunction-associated steatotic liver disease (MASLD), and alcohol-associated liver disease (ALD), contributing to the rising burden of HCC in several previously low-incidence regions, including North America, Australia, and parts of Europe (5, 6). As a result, liver cancer incidence and mortality have increased by 53.7% and 48%, respectively (1, 7).

Given the nature of the disease, diagnosis, staging, and treatment are deemed complex. Therefore, HCC is best approached through a multidisciplinary framework involving hepatologists, radiologists, pathologists, oncologists, and surgeons (8). Curative-intent strategies include liver transplantation (LT), resection, and local ablation, while trans-arterial therapies, radiation therapy, and systemic treatments play key roles in advanced and unresectable tumors (9).

Liver resection (LR) remains a cornerstone of curative-intent treatment for HCC, particularly for solitary tumors in appropriately selected patients with preserved liver function, acceptable portal hypertension-related risk, and adequate future liver remnant (FLR) (10). Over time, minimally invasive liver surgery (MILS), including laparoscopic and robotic approaches, has emerged, sparking debate regarding the optimal surgical approach compared to open surgery (11). While MILS has been associated with reduced blood loss, shorter length of stay (LOS), and lower perioperative morbidity and mortality, widespread adoption remains limited by several barriers, including technical complexity, high procedural costs, restricted instrument availability, the potential need for an experienced bedside assistant surgeon, and limited access to robotic technology (12, 13).

In this review, we discuss the specific challenges associated with LR for HCC and compare open, laparoscopic, and robotic approaches, with emphasis on technical considerations, platform-specific advantages and limitations, clinical scenario-based decision-making, and oncologic outcomes.

This review aims to examine how each approach may be integrated into contemporary HCC management according to tumor anatomy, liver function, technical complexity, and institutional expertise.

## Methods

This narrative review was conceived as a narrative and critical appraisal of the available literature addressing the specific challenges of HCC resection and comparing the outcomes of open, laparoscopic and robotic approaches.

A structured literature search was conducted in PubMed/MEDLINE for articles published between January 2000 and April 2026. The search was last updated on May 15, 2026. The core search strategy combined terms related to HCC, liver resection, and surgical approach, including “hepatocellular carcinoma”, “open”, “laparoscopic”, “robotic”, “minimally invasive”, “liver resection”, and “hepatectomy”, using Boolean operators. Additional targeted searches were performed for specific topics addressed in the review, including cirrhosis, portal hypertension, future liver remnant, liver function assessment, posterosuperior and central liver segments, multifocal disease, recurrent HCC, anatomical resection, and parenchymal-sparing surgery. Reference lists of relevant reviews, consensus statements, guidelines, and selected original studies were also screened to identify additional pertinent publications.

Original studies, comparative cohort studies, case series, systematic reviews, meta-analysis, consensus statements, and international guidelines were considered eligible when they reported technical aspects, perioperative outcomes, oncologic outcomes, or patient-selection considerations relevant to liver resection for HCC. Studies were excluded if they were not published in English, did not specifically address HCC or liver resection, lacked clinically relevant technical or outcome data, or were available only as abstracts, conference proceedings, editorials, or correspondence without original data.

Titles and abstracts were initially screened for relevance to the predefined scope of the review, followed by full-text assessment of potentially eligible publications. Because of the narrative design, no formal quantitative synthesis or systematic risk-of-bias assessment was performed. Study selection prioritized clinical relevance, methodological quality, technical detail, and applicability to the specific scenarios discussed. When multiple studies addressed the same topic, greater weight was given to comparative studies, multicenter analysis, systematic reviews, international guidelines, and technically informative series from experienced hepatobiliary centers.

## Challenges specific to HCC resection

Resection for HCC is frequently burdened by technical and functional constraints that increase operative complexity. Unlike many other hepatic malignancies, HCC often arises in the setting of chronic liver disease, ranging from fibrosis and steatosis to established cirrhosis and, in selected patients, clinically significant portal hypertension (CSPH). Although cirrhosis is not considered an absolute contraindication to LR, current treatment algorithms determine surgical candidacy through integrated assessment of liver function, portal hypertension, tumor burden, planned extent of resection, and FLR adequacy (14, 15). This selective framework reflects the fact that, in HCC, technical feasibility alone does not guarantee postoperative safety.

First, parenchymal transection in a diseased liver is technically demanding. Fibrosis and cirrhosis are associated with a stiffer and less compliant parenchyma, which may reduce liver mobility and make exposure more difficult, particularly for posterior and superior segments. Conversely, steatotic parenchyma may be more fragile and less tolerant of manipulation, making mobilization and transection more delicate. In patients with CSPH, increased venous pressure, the presence of aberrant collateral vessels, and thrombocytopenia may further increase the risk of bleeding and complicate hilar or perihilar dissection.

Second, chronic liver disease reduces the functional reserve of the FLR. The diseased parenchyma has reduced regenerative capabilities, making it more prone to postoperative decompensation and complications (16, 17). Consequently, the FLR cannot be assessed only in volumetric terms but must also be interpreted according to the quality and functional capacity of the underlying parenchyma. This limitation restricts the extent of resection that can be safely performed and reinforces the importance of parenchymal-sparing strategies (18). The issue becomes particularly relevant in patients with multifocal disease, centrally located tumors, or lesions in technically demanding segments, where achieving adequate oncologic clearance may conflict with the need to preserve functional liver tissue.

Finally, the diseased liver remains at risk for both intrahepatic recurrence and *de novo* tumor formation (19). Moreover, liver function may deteriorate after resection, and some patients may progress from compensated to decompensated liver disease, shifting long-term prognosis from a purely tumor-centered perspective to one driven by the underlying hepatic condition (10). Therefore, patient selection, assessment of liver function, and careful definition of the surgical indication are essential to achieve the best balance between oncologic benefit, technical feasibility, and postoperative safety.

These considerations explain why platform selection in HCC should not be based solely on the feasibility of tumor removal, but should be tailored to specific clinical scenarios, tumor location, liver function, and the expected technical demands of the resection.

## Patient selection

Patient selection represents one of the most critical steps in the surgical management of HCC, as it requires simultaneous consideration of tumor-related benefit and liver-related risk (**Figure 1**). The central question is not only whether the tumor is technically resectable, but whether the expected oncologic benefit justifies the functional cost of removing liver parenchyma in a patient who may have chronic liver disease, portal hypertension, impaired regenerative capacity, and a substantial risk of future intrahepatic recurrence. Indeed, resection should be viewed as a balance between two potentially conflicting objectives: achieving oncologically sound tumor clearance and preserving sufficient functional parenchyma to avoid postoperative liver failure (PHLF).

**Figure 1:**
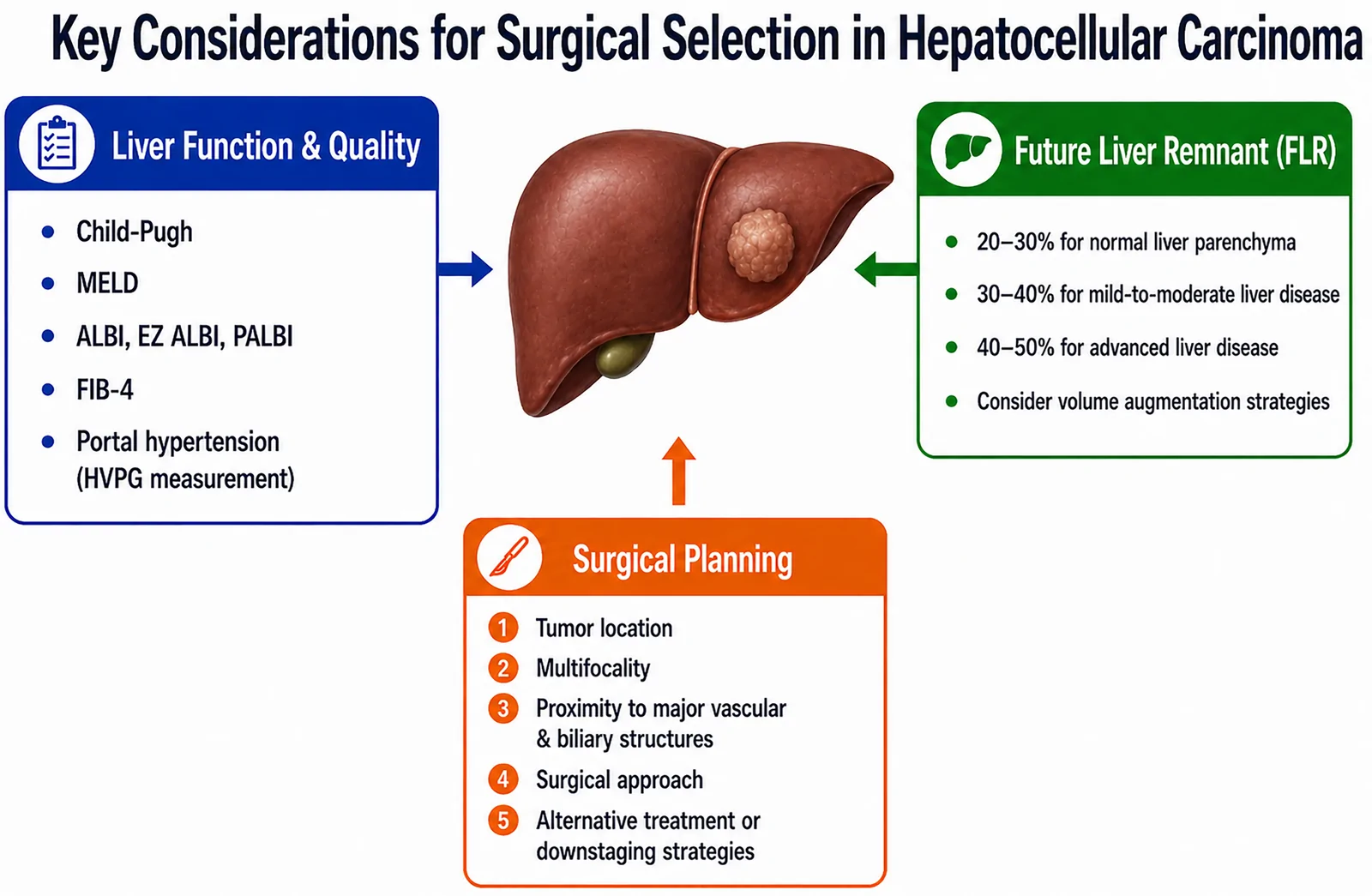
Factors to consider for HCC treatment. Approximate FLR reference ranges; individualized assessment of liver quality, function, portal hypertension, and planned extent of resection is required. MELD, Model for End-Stage Liver Disease; ALBI, Albumin-Bilirubin score; EZ ALBI, Easy Albumin-Bilirubin score; PALBI, Platelet-Albumin-Bilirubin score; FIB-4, Fibrosis-4 score; HVPG, Hepatic Venous Pressure Gradient.

### Liver parenchyma quality and function assessment

The first component of patient selection is the assessment of the underlying liver parenchyma and its functional reserve. HCC commonly arises in the background of chronic liver disease, in which inflammation, steatosis, fibrosis, and cirrhosis may coexist and influence both operative complexity and the safe extent of resection. In this setting, preoperative evaluation should not rely on a single parameter, but should integrate clinical, biochemical, radiological, histological, and hemodynamic information.

Cirrhosis represents the final stage of chronic liver disease and remains a major determinant of operative complexity and postoperative outcomes (20, 21). However, liver parenchyma quality may be impaired even at earlier stages of disease. MASLD has become increasingly prevalent, especially in high-income countries (22). Parenchymal changes range from simple steatosis to steatohepatitis, fibrosis, and ultimately cirrhosis. Nevertheless, extensive steatosis and inflammatory activity may adversely affect postoperative outcomes even before advanced fibrotic transformation occurs (23–25).

Liver function represents another paramount component of preoperative assessment. Several scores and diagnostic tests may be employed to estimate functional reserve and guide the safe extent of resection. Traditionally, the Child-Pugh classification has been used for broad stratification of cirrhotic patients. Child-Pugh C cirrhosis is generally considered a contraindication to LR, whereas in Child-Pugh B cirrhosis surgical candidacy and the extent of resection should be individualized according to functional reserve, portal hypertension, tumor characteristics, and the planned surgical approach (14, 15). Early comparative evidence summarized by Morise et al. suggested that, in patients with HCC and chronic liver disease, laparoscopic LR was associated with reduced postoperative ascites and liver failure compared with open LR, without compromising long-term oncologic outcomes (26). More recently, Troisi et al., in an international multicenter propensity score-matched study of patients with Child-Pugh B cirrhosis, reported lower blood loss, overall morbidity, and major morbidity after laparoscopic compared with open LR, with comparable 90-day mortality and long-term oncologic outcomes (27). Importantly, postoperative morbidity remained higher in patients with portal hypertension and more advanced Child-Pugh B disease, underscoring the importance of careful patient selection (27).

However, even among Child-Pugh A patients, postoperative risk may vary substantially, and additional tools are often required to refine patient selection. The model for end-stage liver disease (MELD) score, originally developed to predict the survival after trans-jugular intrahepatic portosystemic shunt and later adopted for LT allocation, has been shown to predict postoperative morbidity, mortality, and long-term survival after LR for HCC in cirrhotic patients (28–30). Albumin-bilirubin (ALBI) grade has also gained increasing attention because it relies only on two objective laboratory parameters, serum albumin and bilirubin, and avoids the subjective components of the Child-Pugh classification. Several studies have reported an association between worse ALBI grade and adverse short-term outcomes, including post-hepatectomy liver failure, as well as impaired survival after HCC resection (31, 32). Further refinements of ALBI-based assessment have been proposed. EZ (easy) ALBI uses a simplified mathematical formula designed to facilitate bedside calculation (33), whereas platelet-albumin-bilirubin (PALBI) grade incorporates platelet count as an indirect marker of portal hypertension (34). Finally, fibrosis-based indices such as FIB-4 may complement functional assessment by identifying patients with advanced fibrosis and higher liver-related risk. However, FIB-4 should be interpreted primarily as a non-invasive fibrosis/prognostic marker rather than as a direct measure of functional hepatic reserve (35).

Beyond clinical scores, quantitative and dynamic tests may further refine preoperative risk assessment. Indocyanine green (ICG) clearance is a widely used method to evaluate global liver function and guide surgical decision-making in patients with HCC (36). Two parameters are commonly assessed: ICG retention rate at 15 minutes (R15) and ICG plasma disappearance rate (PDR). However, ICG clearance primarily reflects global liver function, without directly accounting for the functional contribution of the FLR. Nuclear medicine-based functional imaging, including technetium-99m galactosyl human serum albumin scintigraphy and technetium-99m mebrofenin hepatobiliary scintigraphy, provide a quantitative measurement of liver function, with the additional advantage of regional functional information (37). These techniques are particularly attractive when major resection is considered in patients with chronic liver disease, although availability, cost, and institutional expertise still limit their widespread adoption.

Taken together, liver functional reserve should be assessed using a multiparametric approach integrating clinical scores, dynamic functional tests, regional functional imaging when available, portal hypertension, and the planned extent of resection, rather than relying on any single measure of operability.

### Portal hypertension evaluation

Portal hypertension is another essential determinant of surgical candidacy. It is classically defined as a hepatic venous pressure gradient (HVPG) greater than 5 mmHg, whereas CSPH is usually defined by an HVPG of at least 10 mmHg. In patients undergoing liver resection for HCC, portal hypertension reflects advanced parenchymal disease and has been associated with increased risk of PHLF, morbidity, and mortality (38–40). HVPG measurement is considered the gold standard for assessing portal hypertension. However, it is invasive in nature; for this reason, several non-invasive surrogates have been proposed, with evidence supporting a fair diagnostic accuracy (41).

Several authors reported favorable outcomes after LR performed with minimally invasive approaches (42–45). Importantly, portal hypertension should not be interpreted as an isolated binary variable, but in relation to liver function, tumor burden, and planned extent of resection. While major hepatectomy in the presence of CSPH remains a high-risk scenario, selected patients with preserved liver function and limited tumor burden may still be considered for minor or parenchymal-sparing resections, particularly in expert centers. In this context, MILS may reduce surgical trauma and postoperative morbidity.

### Future liver remnant adequacy

Another important component is the evaluation of the FLR. In HCC, the adequacy of the remnant liver cannot be judged solely by volume, because the functional quality of the remnant may be impaired by fibrosis, cirrhosis, steatosis, cholestasis, inflammation, or prior treatments. For this reason, preoperative assessment should focus on both the quantity and the quality of the FLR. This distinction is particularly relevant in patients with chronic liver disease, in whom a volumetrically adequate remnant may still be functionally insufficient.

Optimal cutoffs remain debated and should be interpreted according to the condition of the background liver. In patients with normal liver parenchyma, a minimum FLR of approximately 20–30% of the standardized liver volume is generally considered acceptable after major hepatectomy (46–48). Conversely, in patients with injured or diseased liver parenchyma, higher thresholds are required to reduce the risk of PHLF. Cutoffs of at least 30–40% have been proposed for compromised livers (49–51), whereas patients with advanced fibrosis, cirrhosis, metabolic syndrome, or steatohepatitis may require even more conservative thresholds, approaching 40–50%. These values should be considered approximate reference ranges rather than universal thresholds, as the safe FLR depends on the quality and functional capacity of the underlying liver, the planned extent of resection, and the individual patient risk profile.

When the anticipated FLR is insufficient, preoperative volume modulation strategies may be considered. Portal vein embolization (PVE) represents the most established approach to induce hypertrophy of the contralateral liver before major hepatectomy (52). In selected patients with HCC, sequential trans-arterial chemoembolization (TACE) followed by PVE has also been proposed, with the dual aim of controlling tumor progression and promoting FLR hypertrophy. Other strategies, including two-stage hepatectomy or associating liver partition and portal vein ligation for staged hepatectomy (ALPPS), may be considered in highly selected cases, although their applicability in HCC is limited by liver function, portal hypertension, tumor biology, and the risk of progression during the waiting interval.

Hypertrophy-inducing strategies should not be considered only as methods to increase liver volume. The hypertrophic response after PVE may also serve as a functional stress test of the regenerative capacity of the FLR. Insufficient hypertrophy despite technically successful PVE may indicate impaired regenerative potential and identify patients at increased risk of PHLF.

## Clinical scenarios and platform selection

The choice between open, laparoscopic, and robotic LR for HCC should not be interpreted as a simple hierarchy of surgical invasiveness. Rather, platform selection should be viewed as a scenario-specific decision integrating tumor anatomy, liver function, technical complexity, and institutional expertise. This concept is particularly relevant in HCC, where technical resectability alone does not necessarily translate into an appropriate surgical indication. Current treatment algorithms support resection as the treatment of choice for localized HCC in the absence of cirrhosis, while recommending a more selective approach in cirrhotic patients with limited tumor burden, well-compensated liver disease, absence of a favorable portal hypertension-related risk profile, and adequate FLR. Minimally invasive approaches may further reduce perioperative morbidity and enhance recovery, but their role must be interpreted within the specific clinical and technical context.

Accordingly, the following section discusses platform selection across distinct scenarios, including small solitary tumors, posterosuperior and central tumors, multifocal disease, and recurrent HCC (**Table 1**).

**Table 1:** Scenario-based platform strategy for liver resection in HCC.

Clinical scenario	Main surgical priority	Platform-oriented strategy	Key caution
Small solitary nodule	Local tumor control, minimizing morbidity	MILR is favored. LLR is generally appropriate for superficial/peripheral tumors; RLR may be useful for small lesions in technically challenging locations	Individualized treatment: surgery vs percutaneous ablation strategies vs LT
Posterosuperior location	Improve exposure, maintain orientation, control bleeding, and preserve parenchyma in segments 7/8	MILR feasible in expert centers. RLR may be favored by improved maneuverability. OLR remains appropriate for large, deep, vascularly complex, or oncologically uncertain lesions	Evidence mostly retrospective and expertise-dependent; PS location remains a marker of technical complexity and conversion risk.
Central/deep location	Define a safe transection plane close to major veins, Glissonean pedicles, hilar plate, or hilum while avoiding excessive parenchymal sacrifice	OLR remains highly relevant when major vascular/biliary control or complex central resection is anticipated. LLR is feasible only in selected cases and expert centers. RLR may serve as a technical enabler for selected parenchymal-sparing resections around major vessels	Robotic parenchymal-sparing strategies for central/deep tumors remain rarely reported; robotics should not be considered an established standard approach
Multifocal disease	Maximize meaningful tumor control while minimizing functional liver loss	Platform choice should follow tumor distribution. OLR allows for wide exploration, bilobar disease, multiple resections, or combined procedures. LLR may be appropriate for limited superficial lesions. RLR may be useful for complex parenchymal-sparing resections involving deep, central, or PS locations	The main issue is biological and functional selection, not platform feasibility. Consider LT eligibility
Recurrent HCC	Achieve repeat tumor clearance despite adhesions, altered anatomy, and reduced liver reserve	Repeat resection is valuable in selected patients. LLR may reduce blood loss, complications, and recovery time in selected recurrent tumors. RLR is promising but evidence remains limited. OLR remains important for severe adhesions, distorted anatomy, or complex vascular control	Most evidence comes from selected cohorts, often Asian series; transferability to Western populations and the specific role of RLR remain uncertain

HCC, hepatocellular carcinoma; MILR, minimally invasive liver resection; OLR, open liver resection; LLR, laparoscopic liver resection; RLR, robotic liver resection; LT, liver transplantation; PS, posterosuperior.

### Small solitary nodule

Solitary HCC represents the most established indication for LR in non-cirrhotic patients. In cirrhotic patients, however, LR is most strongly supported in those with limited tumor burden, well-compensated liver disease, adequate FLR, and a favorable portal hypertension-related risk profile. In this setting, a 5-year survival exceeding 70% and a below 3% postoperative mortality after LR is reported (14).

For early-stage solitary HCC, particularly tumors ≤3 cm, the optimal curative strategy remains debated. Locoregional treatments, especially thermal ablation with radiofrequency ablation (RFA) or microwave ablation (MWA), have been proposed as alternative strategies to LR. In a recent network meta-analysis focused on solitary HCC ≤3 cm, Kim et al. reported greater 3-year and 5-year overall survival (OS) and recurrence-free survival (RFS) after LR compared to RFA. Conversely, no significant differences in terms of 3-year and 5-year OS were observed between LR and MWA or between LR and TACE, although MWA was associated with significantly lower 3-year and 5-year RFS compared to LR (53). However, subsequent randomized controlled trials comparing LR and RFA reported increased morbidity in the LR groups without significant benefits in terms of OS and RFS (54, 55). Radiation-based local therapies, including transarterial radioembolization (TARE) and external beam radiotherapy (EBRT), have also been increasingly investigated in selected early-stage patients, particularly in those unsuited for surgery or where a high risk of recurrence is expected after ablation (15). Therefore, the optimal treatment strategy for very early and early-stage solitary HCC remains controversial and should be individualized according to tumor size, location, liver function, expected margin, and patient fitness.

Within the surgical arm, the ideal operative approach is also debated. Two large propensity score-matched (PSM) studies compared open, laparoscopic, and robotic LR for early-stage HCC, including patients with BCLC stage 0 or A (11, 56). Both studies reported longer operative times and shorter LOS for minimally invasive approaches compared to open. Zhu et al. found no additional significant differences in terms of postoperative complications, OS, and disease-free survival (DFS) among the groups (56). Xia et al. also reported a significantly lower rate of postoperative complications in the robotic group compared to the others. In the same study, robotic LR was also associated with the lowest complication rates and the highest quality-of-life scores compared to laparoscopic and open groups. Laparoscopic LR showed less postoperative complications and higher quality-of-life scores compared to open, while also providing the most favorable cost-effectiveness profile (11).

Taken together, these data support the feasibility and potential perioperative benefits of minimally invasive approaches in selected patients with early-stage HCC. This is particularly relevant because, in small solitary tumors, LR is often weighed against less invasive percutaneous strategies. By reducing surgical trauma, laparoscopic and robotic approaches may narrow the invasiveness gap between LR and percutaneous ablation, potentially making resection more attractive for solitary HCCs in anatomically favorable or technically manageable locations. Conversely, percutaneous ablation remains a valuable option for patients with very small tumors, limited hepatic reserve, high operative risk, or lesions in unfavorable locations for which LR would require disproportionate parenchymal sacrifice.

### Posterosuperior location

Tumors involving posterosuperior (PS) segments (i.e., segments 7 and 8) represent a technically demanding scenario for LR. Initially, open LR was the mainstay of treatment, as laparoscopic LR was traditionally considered technically challenging and generally limited to selected cases due to the tumor’s challenging location and the relationship with the hepatic veins and Glissonean pedicles (57, 58). However, with the progressive adoption of MILS and refinements in surgical technique, laparoscopic management of PS lesions has become increasingly feasible in experienced centers. Several technical adaptations have been proposed to improve exposure, including tailored port placement, intercostal trocars, and modifications in patient positioning (59–61). Morise et al. described a caudal approach using the left lateral position for posterior sectionectomy and the semi-prone position for S7 segmentectomy and partial resections of S7 and deep S6, exploiting gravity-assisted displacement of the liver and adjacent viscera to improve exposure of the posterosuperior area (62). Xiao et al. reported that laparoscopic LRs of PS lesions were as safe and effective as conventional open LR, with reduced blood loss, postoperative complications, and hospital stay (63). Similarly, Scuderi et al. showed reduced postoperative complication rates, LOS, and duration of required analgesia in the laparoscopic group compared to a PSM open LR group (64). Nonetheless, some authors reported that LR involving the PS segments was an independent predictive factor for conversion to open surgery, with a 12.7% conversion rate in the PS group in comparison to 2.5% for tumors involving other segments (65).

In this context, robotic LR may offer specific technical advantages compared with conventional laparoscopy, including greater instrument articulation, stable three-dimensional visualization, and integrated intraoperative imaging with ultrasound and ICG fluorescence guidance, all of which may facilitate precise dissection in these anatomically challenging segments (66, 67). Of note, an international retrospective PSM analysis showed a decrease in blood loss ≥ 500 ml (23.9% vs 48.8%, p=0.001), intraoperative transfusion (10.2% vs 23.9%, p=0.014), and open conversion (2.3% vs 11.4%, p=0.016) when comparing robotic and laparoscopic resection of PS segments, respectively (68). This was further corroborated by Krenzien et al., reporting similar findings when comparing robotic and laparoscopic limited LR for PS tumors (69).

Moreover, although anatomical LR has shown oncological benefits over non-anatomical LR (70), it is rarely performed for PS segment tumors, due to the technically demanding nature of anatomical resections using the minimally invasive approach (71). A retrospective cohort analysis by Lee et al. compared between laparoscopic anatomical and non-anatomical resections for PS HCC tumors. While non-anatomical resections result in better perioperative outcomes, both approaches provide comparable 1-, 3-, and 5- year OS and RFS (72). Additionally, recurrence following non-anatomical resection was more commonly intrahepatic, potentially due to remnant hepatic ischemia promoting tumor growth, whereas recurrence after anatomical resection was more frequently extrahepatic, possibly related to greater intraoperative blood loss resulting in tumor spillage and hematogenous dissemination, or impaired antitumor immunity due to high systemic inflammation (72–74). To perform anatomical resection of these segments, a trans-parenchymal approach or the ICG staining techniques are usually employed (66, 75). Alternatively, the ultrasound-guided finger compression of the tumor-feeding portal pedicle has been established in open surgery (76). The same technique has also been described in the minimally invasive setting, although its adoption is limited by technical complexity (77).

Management of HCC involving the PS liver segments remains technically demanding because of the complex anatomy, difficult surgical exposure, and steep learning curve associated with these lesions. Nevertheless, continuous advances in MILS, including refinements in laparoscopic and robotic techniques, improved visualization platforms, fluorescence-guided surgery, and growing surgical expertise, have substantially expanded the feasibility and safety of laparoscopic and robotic LR for PS tumors.

### Central or deep location

Central or deeply located HCCs represent a distinct technical scenario because of the proximity to the major hepatic veins, Glissonean pedicles, the hilar plate, or the hepatic hilum. In contrast to superficial peripheral tumors, for which limited wedge resection or ablation may be technically straightforward, central and deep lesions require precise preoperative mapping and intraoperative navigation to balance oncologic clearance with preservation of functional parenchyma. This balance is particularly relevant in patients with chronic liver disease, in whom excessive parenchymal sacrifice may increase the risk of postoperative liver failure. Hence, tumor location is typically recognized as a key determinant of treatment selection.

The main surgical challenge is to define a safe transection plane that achieves tumor clearance without unnecessary sacrifice of vascular inflow, venous drainage, or biliary structures. For lesions adjacent to the major vascular and biliary structures, minor errors in spatial orientation may translate into bleeding, bile leak, venous congestion, or conversion to a larger resection than initially planned. In this setting, intraoperative ultrasound is essential to confirm tumor depth, define its relationship with major vessels, detect additional lesions, and guide the transection plane (78). Fluorescence-guided techniques, particularly indocyanine green-based positive or negative staining, may further assist anatomical or subsegmental resection (79, 80).

Platform selection in central/deep HCC should therefore be tailored to the expected need for exposure, vascular control, and fine parenchymal dissection. Open surgery remains highly relevant when the lesion is large, close to the hilum or major hepatic veins, associated with suspected vascular or biliary involvement, or when a major hepatectomy or complex central resections are anticipated. Its advantages include tactile feedback, direct vascular control, flexible exposure, and rapid bailout in case of bleeding. Although technical feasibility of laparoscopic LR for central or deeply located tumors has been reported, these procedures remain among the most demanding applications of conventional laparoscopy (81–83). Accordingly, central and deep tumors have not traditionally represented standard indications for laparoscopic LR in most centers, and their minimally invasive management is generally limited to highly experienced units (84). In this context, robotic surgery may offer potential advantages through stable three-dimensional visualization, wristed instrumentation, improved ergonomics, and enhanced suturing capability, which may facilitate precise parenchymal-sparing dissection around major vessels (85). However, robotic parenchymal-sparing strategies for tumors located in these locations are rarely reported (86, 87). Therefore, the role of robotics in central/deep HCC should be interpreted as a potential technical enabler in selected cases, rather than as an established standard approach.

### Multifocal disease

Multifocal HCC represents one of the most controversial scenarios for LR, because tumor multiplicity may reflect either intrahepatic metastatic spread from the index tumor or multicentric carcinogenesis within a chronically diseased liver. Consequently, the decision to resect should not be based solely on technical feasibility, but on the interaction between tumor burden, distribution of nodules, liver function, portal hypertension, transplant eligibility, and the availability of alternative locoregional strategies. In this setting, platform selection is secondary to the more fundamental question of whether resection offers a meaningful oncologic benefit without excessive functional cost.

For patients with multifocal HCC falling within accepted transplant criteria, liver transplantation remains conceptually attractive because it treats both the tumor burden and the underlying carcinogenic liver disease. However, transplant eligibility is not defined by tumor burden alone and depends on biological selection, alpha-fetoprotein levels, response to bridging or downstaging therapy, comorbidities, allocation rules, donor availability, and local policy.

When transplantation is not feasible, the optimal strategy remains debated. A recent multicenter cohort study by Vitale et al. including 720 patients with early multinodular HCC reported higher 1-, 3-, and 5-year OS after LR compared with RFA and TACE, suggesting that LR may provide a survival advantage in carefully selected patients (88). Similarly, in a comparative retrospective study between LR for unifocal and multifocal HCC, Yu et al. found no significant differences in terms of OS and DFS (89).

The evidence becomes even more complex in multifocal disease beyond conventional early-stage criteria or corresponding to intermediate-stage HCC. Several studies have suggested that LR may be associated with better survival than TACE in selected patients with technically resectable intermediate-stage disease. A randomized trial by Yin et al. compared open partial hepatectomy with TACE for resectable multiple HCC beyond Milan criteria, supporting the possibility that surgery may benefit a subset of patients with resectable multifocal disease (90). Similarly, meta-analysis comparing hepatic resection with TACE in intermediate-stage (BCLC-B) HCC have suggested improved survival with LR in selected cohorts (91, 92). Nevertheless, these comparisons remain intrinsically vulnerable to indication bias: patients offered surgery are generally those with preserved liver function, lower tumor burden, favorable anatomy, and better performance status. Therefore, the apparent survival advantage of resection should not be generalized to all patients with multifocal HCC.

From a technical standpoint, multifocal HCC shifts the surgical objective from removing a single lesion to designing the least functionally costly strategy capable of achieving meaningful tumor control. If nodules are confined to one lobe or one anatomical territory, anatomical resection or even major hepatectomy may be considered, provided that liver function and FLR are adequate. Conversely, in bilobar or scattered disease, multiple limited resections, parenchymal-sparing segmentectomies, or combined resection and ablation may be more appropriate to avoid disproportionate parenchymal sacrifice. In this scenario, intraoperative ultrasound is essential to confirm tumor number, define relationships with vascular structures, identify occult lesions, and adapt the operative plan. For tumors involving multiple segments or when oncologic benefit is uncertain, bridging or downstaging therapies may also provide a useful “test of time” before committing to LR (15).

The role of minimally invasive surgery in multifocal HCC should therefore be interpreted through the lens of parenchymal preservation rather than simple reduction of surgical trauma. Laparoscopy may be suitable for selected patients with limited, superficial, or anterolateral multifocal disease, especially when multiple minor resections can be performed safely. Robotic surgery may offer potential advantages when technically demanding parenchymal-sparing resections are required, particularly for deep, central, or posterosuperior nodules, where wristed instrumentation, stable three-dimensional visualization, and suturing capability may facilitate precise dissection and hemostasis. Open surgery remains relevant when multifocal disease requires wide exploration, complex intraoperative mapping, multiple resections across different segments, or combined procedures.

Multifocal HCC remains strongly driven by tumor biology and recurrence risk, and surgical treatment should be reserved for patients in whom tumor distribution, liver function, and expected FLR make resection oncologically meaningful and functionally sustainable. In many patients, especially those with diffuse bilobar disease, clinically significant portal hypertension, limited hepatic reserve, or clear transplant eligibility, non-resection strategies or transplantation may represent more appropriate options.

### Recurrent tumors

Despite the availability of a myriad of curative treatments, including LR, thermal ablation, and LT, long-term outcomes of HCC are still affected by a high recurrence rate, reaching up to 18% and 70% after LT and LR, respectively (93, 94). Early recurrence (i.e., within 2 years from treatment) is linked to tumor-related factors, such as size, multifocality and microvascular invasion, whereas late recurrence is related to underlying liver disease (93). Given the pivotal impact of HCC recurrence on long-term survival, a thorough understanding of the factors that predispose to recurrence, as well as the selection of the most effective therapeutic approach for recurrent tumors, is essential for improving oncologic outcomes.

The ideal approach to recurrent HCC remains under debate (15). While locoregional therapies such as TACE are frequently employed for recurrent HCC (95), repeat surgical resection remains a valuable option in carefully selected patients with resectable disease and adequate liver reserve (96). Repeat LR comes with its own set of challenges, including the presence of adhesions and altered normal anatomy of the liver (97). Moreover, most experience regarding safety and oncological benefit of re-resection comes from Asian cohorts and is yet to be unconditionally transferable to Western patients (98). In their multicentric study, Famularo et al. reported higher 1-,3-, and 5-year survival when comparing curative repeat LR (98.3%, 76.7%, 63.8%, respectively) and palliative use of TACE with or without sorafenib (91.7%, 64.2%, 48.9%) (99).

Due to the complexity of these procedures, these resections have traditionally been performed via the open approach. However, major advancements in surgical technique and technology increase the utilization of MILS for recurrent HCC (100). Several comparative studies have associated laparoscopic repeat LR with reduced blood loss, fewer postoperative complications, and faster postoperative recovery (101, 102). While some studies reported longer disease-free survival and favorable short-term outcomes (103), others showed comparable survival (104). Among the largest comparative experiences, Morise et al. reported an international multicenter propensity score-based analysis of 1,582 repeat liver resections for HCC performed across 42 centers. After matching, laparoscopic repeat LR was associated with lower intraoperative blood loss but longer operative time compared with the open approach, while other short-term outcomes and long-term OS and DFS were comparable between groups (105). In a subsequent subgroup analysis of the same international cohort, Miyama et al. focused on technically less complex repeat resections, defined by tumors located more than 1 cm from major Glissonean pedicles and hepatic veins. After propensity score matching, laparoscopic repeat LR was associated with lower blood loss and postoperative morbidity, while operative time and long-term survival were comparable with open repeat LR (106). These findings suggest that the perioperative benefits associated with laparoscopy may be particularly evident in appropriately selected repeat resections of lower technical complexity. The role of robotic surgery in the management of recurrent disease remains to be established, owing to the relative paucity of published literature in this setting. Further prospective studies and large-scale multicenter retrospective analyses are needed to better characterize its safety, feasibility, oncologic efficacy, and potential advantages over open and laparoscopic surgical approaches in patients with recurrent disease.

## Comparative outcomes of open, laparoscopic, and robotic resection

Over the past two decades, the surgical management of HCC has undergone a substantial evolution, with MILS increasingly challenging the historical dominance of open LR. As a result, an expanding body of literature has compared open, laparoscopic, and robotic LR with respect to perioperative safety, postoperative recovery, oncologic adequacy, recurrence patterns, and long-term survival. While minimally invasive approaches have consistently demonstrated advantages in blood loss, postoperative morbidity, and hospital LOS, important questions remain regarding the relative benefits of robotic versus laparoscopic surgery, the impact of surgical approach on margin status and recurrence, and whether these short-term advantages translate into improved long-term outcomes (107). The following section reviews the available comparative evidence on open, laparoscopic, and robotic LR for HCC, focusing on perioperative outcomes, oncologic efficacy, and the evolving role of each approach in contemporary hepatobiliary practice.

A retrospective analysis of the American College of Surgeons National Surgical Quality Improvement Program (ACS-NSQIP) hepatectomy database comparing minimally invasive liver resection (MILR) and open LR demonstrated that MILR independently reduced postoperative morbidity after adjustment for patient factors, tumor stage, and liver characteristics. Importantly, this study identified open surgery itself as an independent predictor of postoperative complications (OR 1.42), highlighting that surgical approach may influence outcomes beyond differences in patient selection (108).

Additionally, several institutional PSM studies subsequently expanded the discussion beyond short-term outcomes. Goh et al. combined both propensity score matching and coarsened exact matching while controlling tumor stage, vascular invasion, multifocality, tumor grade, and cirrhosis. Although overall and recurrence-free survival were similar between approaches, minimally invasive surgery was associated with a significantly longer median time-to-recurrence (70 vs. 40.3 months), suggesting a potential oncologic benefit that warrants further investigation (109).

Similarly, Wong et al. formulated a single-center comparative analysis that incorporated the IWATE difficulty score into their matching criteria, thereby accounting for technical complexity. This study demonstrated that reduced blood loss, blood transfusions, and postoperative complications were advantages that persisted in the MILR cohort even after controlling for operative difficulty (110). This further supported the applicability of MILR across a broad range of technical scenarios. Park et al. expanded on these observations through a decade-long experience from a tertiary transplant center in which MILR was performed in a patient population >60 years of age and yet still achieved comparable oncologic outcomes to open LR. Importantly, despite an older age in the minimally invasive cohort, overall survival and disease-free survival remained equivalent, while hospital stay and operative duration were reduced (111). The authors further emphasized the potential role of MILR in expanding resection eligibility among elderly patients and highlighted the potential advantages of reduced adhesions and improved feasibility of future salvage treatments following recurrence. This study therefore provided important evidence supporting the safety of MILR in an aging HCC population while preserving long-term oncologic efficacy.

The impact of MILR in biologically aggressive disease was specifically addressed by Yang et al., who evaluated only patients with microvascular invasion-positive HCC. This study is unique because it challenged the concern that MILR might compromise oncologic principles in high-risk tumors. Importantly, regardless of anatomical resection status, surgical margin width, IWATE difficulty score, or use of postoperative adjuvant therapy, no significant differences in recurrence-free and overall survival were observed between MILR and open LR (112). These findings suggest that MILR does not appear to compromise oncologic radicality even in one of the highest-risk pathological subgroups encountered in HCC surgery.

Several studies also explored more nuanced oncologic outcomes beyond traditional survival metrics. Birgin et al., using the National Cancer Database to compare open LR vs MILR, reported a paradoxical finding in which MILR was associated with higher positive-margin rates yet equivalent long-term survival (113). This observation challenged the traditional assumption that margin positivity necessarily translates into inferior outcomes and remains one of the most important cautionary findings in the minimally invasive HCC literature. In contrast, the nationwide multicenter study comparing robotic, laparoscopic and open approaches by Xia et al. demonstrated that although recurrence-free and overall survival were also similar among all approaches, MILR was also associated with more favorable recurrence patterns, including lower rates of aggressive recurrence beyond Milan criteria, lower advanced-stage recurrence, and greater opportunities for repeat curative treatment (11). These findings suggest that recurrence biology and recurrence treatability may represent more sensitive oncologic factors than overall survival alone.

The role of robotic surgery has also evolved considerably. Di Benedetto et al. reported the largest western multicenter robotic HCC experience and demonstrated a significant reduction in PHLF compared with open surgery (114). This finding is particularly important because liver failure remains one of the most clinically relevant complications following hepatectomy and may be especially important in cirrhotic patients. Similarly, the single-institutional analysis by Kato et al. highlighted the feasibility of robotic surgery in technically demanding settings, reporting a high proportion of complex anatomic resections, and more than one-third of cases performed in the setting of repeat hepatectomy (115). The exceptionally low conversion rate (approximately 1%) observed in this series suggests that robotic technology may be particularly valuable in complex scenarios.

Direct robotic versus laparoscopic comparisons have yielded more nuanced conclusions. A single-institutional analysis by Liu et al. demonstrated improved bleeding control during robotic major hepatectomy, whereas operative time remained longer, and most postoperative outcomes were comparable (116). Likewise, the International Multicentric Minimally Invasive Liver Resection (SIMMILR-5) study showed that many of the perioperative advantages historically attributed to robotic surgery were achieved by advanced laparoscopic surgery, with robotic approaches failing to demonstrate clear superiority (117). These findings emphasize that robotic surgery should not necessarily be viewed as a replacement for advanced laparoscopy but rather as a complementary platform that may provide the greatest benefit in highly complex resections and difficult anatomic locations.

Finally, Park et al. provided an important historical perspective through a twenty-year change-point analysis of textbook outcomes after HCC resection. Rather than comparing surgical approaches directly, the authors demonstrated that the widespread adoption of minimally invasive and parenchymal-sparing strategies coincided with dramatic improvements in operative efficiency, transfusion rates, postoperative morbidity, length of stay, and overall textbook outcome achievement (118). This study suggests that the evolution of modern HCC surgery is not attributable solely to a change in surgical platform but rather to a broader paradigm shift toward minimally invasive, precision-based, and parenchymal-preserving liver surgery.

## Discussion

Taken together, the available literature consistently demonstrates that MILS may be associated with favorable perioperative outcomes compared with open LR for patients with HCC, while maintaining comparable oncologic efficacy (**Table 2**). This conclusion is supported not only by multiple PSM institutional and national database studies, but also by the recent network meta-analysis by Abdelsamad et al., which represents the highest level of evidence among the available comparative data. In that analysis, robotic LR was associated with significantly fewer overall complications (OR 0.52, 95% CI 0.35–0.77) and major complications (OR 0.44, 95% CI 0.28–0.67) than open LR, lower blood transfusion requirements (OR 0.53, 95% CI 0.32–0.88), reduced blood loss (MD −144.6 mL), and shorter hospital stay (MD −3.0 days), while maintaining equivalent R0 resection rates and long-term survival outcomes. Laparoscopic LR similarly demonstrated lower overall complications (OR 0.64, 95% CI 0.48–0.85), lower major complications (OR 0.56, 95% CI 0.33–0.94), reduced blood loss (MD −93.4 mL), and shorter hospitalization (MD −2.4 days) compared with open LR (107).

**Table 2:** Summary of comparative evidence for open, laparoscopic, and robotic LR for HCC (107–117).

Outcome	Minimally invasive vs open LR	Robotic vs laparoscopic LR	Interpretation
Blood loss	Lower blood loss is reported in several matched studies and in the NMA (107, 112)	NMA and a study focused on major hepatectomy reported lower blood loss with RLR; other direct comparisons reported similar results (107, 116, 117)	Consistent advantage of MILS vs OLR; possible RLR advantage vs LLR but not uniformly reproduced
Transfusion requirement	Lower transfusion requirements have been reported in selected matched studies and pooled analysis (107, 108, 110)	No consistent difference demonstrated in the available direct comparisons	Evidence favors MILS over OLR, while a platform-specific advantage between RLR and LLR is not established
Operative time	Results are heterogeneous: similar, longer, or shorter operative times with MILS have been reported depending on study and case mix (108–111, 117)	RLR is frequently associated with longer operative time; this was demonstrated in the NMA and in major-hepatectomy comparisons (107, 116, 117)	No consistent MILS-vs-OLR effect; longer operative duration remains a recurrent finding for RLR vs LLR
Length of stay	Shorter LOS after MILS is one of the most consistent findings across matched studies and pooled evidence (107–111, 113, 114, 117)	Most direct comparisons show similar LOS, whereas SIMMILR-5 reported longer LOS after RLR than LLR (115–117)	Consistent advantage of MILS vs OLR; no evidence of a consistent RLR advantage over LLR
Overall morbidity	Lower morbidity after MILS is supported by multiple matched studies and the NMA (107–110)	NMA found no significant difference between RLR and LLR; direct comparative studies generally report comparable morbidity (107, 115–117)	MILS is associated with lower morbidity vs OLR; current evidence does not establish a hierarchy between RLR and LLR
Major complications	Pooled evidence suggests fewer major complications with both LLR and RLR vs OLR; individual studies are less consistent (107, 110)	Overall difference is not consistently demonstrated. An exploratory NMA subgroup suggested a possible RLR benefit in major hepatectomy (107, 116)	Potential MILS advantage vs OLR. Any RLR advantage in complex/major resections remains exploratory
PHLF	Evidence is heterogeneous. A multicenter PSM study reported lower PHLF after RLR than OLR, whereas other studies found no significant difference (108, 114)	Insufficient evidence for a consistent difference (107, 116)	A potential reduction after MILS/RLR has been reported, but pooled evidence does not establish a definitive platform-specific advantage
Resection margin/R0	Generally comparable; however, one large NCDB PSM study reported a higher positive-margin rate after MILR (6% vs 4%) (107, 113, 117)	No significant difference in pooled or most direct comparisons (107, 115–117)	Oncologic radicality appears broadly comparable, but the discordant NCDB finding warrants caution
Overall survival	Available adjusted studies generally report comparable OS (109–114)	Available HCC-specific comparisons report comparable OS (107, 115)	No survival advantage has been established for any platform
DFS/RFS	Generally comparable. Goh reported longer time-to-recurrence after MILR in PSM but not CEM analysis (109, 110, 112)	Available HCC-specific comparisons report comparable DFS/RFS (107, 115)	Long-term disease control appears broadly comparable; isolated favorable findings should be considered hypothesis-generating
Conversion to open surgery	Applicable only to minimally invasive approaches	NMA reported a lower conversion rate with RLR than LLR; individual series are heterogeneous and strongly expertise-dependent (107, 116, 117)	RLR may be associated with lower conversion in pooled data, but institutional experience and case selection are important confounders

HCC, hepatocellular carcinoma; LR, liver resection; MILS, minimally invasive liver surgery; OLR, open liver resection; LLR, laparoscopic liver resection; RLR, robotic liver resection; NMA, network meta-analysis; LOS, length of stay; PHLF, post-hepatectomy liver failure; OS, overall survival; DFS, disease-free survival; RFS, recurrence-free survival; PSM, propensity-score matching; CEM, coarsened exact matching.

These findings mirror the observations of several clinical studies performed in differing populations, several of which reported reduced perioperative morbidity, shorter LOS, and lower transfusion requirements with MILS approaches while preserving RFS and OS (108–112, 114, 115). Among minimally invasive techniques, laparoscopy remains the more established and widely validated platform, whereas robotics may provide technical advantages in selected complex resections. Abdelsamad et al. also demonstrated significantly lower conversion rates with robotics compared with laparoscopy (OR 0.45, 95% CI 0.26–0.78), reduced blood loss (MD −51.2 mL), and a trend toward fewer complications, with robotic surgery ranking first in 8 of 11 evaluated outcomes. Importantly, exploratory subgroup analysis suggested that the robotic advantage may be amplified in major hepatectomy, where robotic surgery reduced major complications by 66% compared with open resection (OR 0.34, 95% CI 0.14–0.84) and demonstrated lower complication rates than laparoscopy (OR 2.99 favoring robotics) (107). These exploratory findings raise the hypothesis that some technical features of the robotic platform may become increasingly relevant as procedural complexity increases; however, this remains to be confirmed in adequately controlled comparative studies.

Nevertheless, several direct comparative studies, including SIMMILR-5, the robotic major hepatectomy series, and the Japanese robotic audit, found broadly equivalent oncologic and perioperative outcomes between advanced laparoscopic and robotic LR, with robotics often associated with longer operative duration (115–117). The principal cautionary finding remains the NCDB analysis, which identified higher positive margin rates after minimally invasive LRs despite unchanged long-term survival (113). Finally, the longitudinal TO analysis demonstrated that the progressive adoption of minimally invasive and parenchymal-sparing hepatectomy over the last two decades has paralleled substantial improvements in operative efficiency, blood loss, transfusion requirements, postoperative morbidity, and TO achievement (118).

Beyond perioperative and oncologic outcomes, the broader implementation of robotic liver surgery is influenced by several practical and institutional factors. Robotic hepatectomy requires dedicated training and progression along a substantial learning curve, and outcomes reported by highly experienced teams may not be immediately reproducible during the development of new programs. Capital investment, maintenance and procedural costs, operating-room logistics, and limited access to robotic platforms may further restrict widespread adoption. Moreover, surgeon and center volume are likely important modifiers of perioperative outcomes, although these factors are inconsistently reported or accounted for in comparative studies. Therefore, the potential technical advantages of robotics should be interpreted in relation to institutional resources, team experience, and procedural volume, and outcomes from high-volume referral centers should not be extrapolated uncritically to lower-volume settings (12, 85).

Overall, the cumulative evidence supports minimally invasive hepatectomy as a feasible and safe strategy in appropriately selected patients with HCC. Laparoscopy currently has the most mature evidence base, while robotics should be viewed as a complementary platform whose potential benefits in complex resections, major hepatectomy, and anatomically challenging tumors require further validation.

## Limitations

This review has several limitations that should be considered when interpreting its findings. To begin with, the literature search was conducted using a single database, which may have resulted in the omission of relevant studies and limits the comprehensiveness of the evidence base. Second, a formal risk-of-bias assessment was not performed; therefore, the methodological quality and potential sources of bias within individual studies could not be systematically quantified. In addition, the restriction to English-language publications may have resulted in the exclusion of relevant evidence published in other languages. Furthermore, although technically informative reports from experienced hepatobiliary centers were particularly valuable for describing complex laparoscopic and robotic strategies, their inclusion may overrepresent outcomes achievable in highly specialized settings and may therefore limit the generalizability of some technical conclusions.

Third, much of the available comparative evidence consists of retrospective observational studies. These studies are inherently susceptible to selection bias and confounding, particularly when comparing open, laparoscopic, and robotic approaches. Consequently, observed differences in perioperative or oncologic outcomes should not be interpreted as evidence of a definitive superiority of one surgical approach over another. Moreover, substantial heterogeneity exists among studies with respect to patient selection, tumor burden and location, definitions of postoperative and oncologic outcomes, surgical techniques, and duration of follow-up, limiting direct comparison across studies.

Finally, robotic liver surgery remains concentrated in experienced, high-volume centers, where outcomes may reflect advanced surgeon expertise, institutional resources, and established learning curves. This concentration may limit the generalizability of reported robotic outcomes to lower-volume centers or institutions with less extensive experience. These limitations should be considered when interpreting the apparent advantages of minimally invasive approaches and underscore the need for prospective multicenter studies and standardized outcome definitions to better establish the comparative effectiveness and broader applicability of different LR strategies for HCC.

## Conclusions

The role of LR in HCC has evolved substantially, supported by improvements in patient selection, surgical planning, perioperative management, and minimally invasive surgical technology. These advances have contributed to the application of LR beyond selected patients with small solitary tumors, including carefully selected patients with multifocal or recurrent HCC and tumors in technically challenging locations, such as the posterosuperior and central liver. However, the evidence supporting these expanded indications, and their chosen approach, remains heterogeneous and is influenced by patient selection, tumor characteristics, liver function and reserve, operative complexity, surgeon experience, and institutional expertise.

Open LR remains an important approach for complex resections, while laparoscopic and robotic techniques appear to provide comparable oncologic outcomes in appropriately selected patients, with potential advantages in perioperative recovery. Robotic surgery may offer technical and ergonomic advantages in selected anatomically difficult procedures; however, whether these translate into clinically meaningful long-term benefits over laparoscopic or open approaches remains uncertain.

Further prospective multicenter studies and randomized trials are needed to better establish the comparative effectiveness of open, laparoscopic, and robotic approaches across different HCC populations. Future research should also evaluate the integration of surgery with systemic and locoregional therapies, including contemporary immunotherapy-based and targeted treatment strategies, particularly in patients at high risk of recurrence. More robust evidence is required to clarify the long-term oncologic outcomes, cost-effectiveness, learning curve, and generalizability of robotic liver surgery, particularly outside expert high-volume centers. Ultimately, individualized multidisciplinary treatment strategies will be essential to optimize patient selection and balance oncologic benefit with preservation of hepatic function.
